# Characterization of the complete chloroplast genome of the ornamental plant *Osmanthus cooperi*

**DOI:** 10.1080/23802359.2019.1627951

**Published:** 2019-07-11

**Authors:** Xiaofei Wang, Fuyue Cai, Cheng Zhang, Min Zhang, Yongfu Li, Yifan Duan

**Affiliations:** a Department of Bioengineering, Huangshan Vocational and Technical College, Huangshan, Anhui, China;; b Nanjing Foreign Language School, Nanjing, Jiangsu, China;; c International Cultivar Registration Center for Osmanthus, College of Biology and the Environment, Nanjing Forestry University, Nanjing, Jiangsu, China;; d Co-Innovation Center for Sustainable Forestry in Southern China, Nanjing Forestry University, Nanjing, Jiangsu, China

**Keywords:** Oleaceae, ornamental, phylogeny, genome

## Abstract

*Osmanthus cooperi* is an evergreen ornamental plant belonging to the olive family. In this study, its complete chloroplast genome was assembled from the whole genome Illumina sequencing data. The circular genome is 155,262 bp long, and comprises a pair of inverted repeat regions (IRs, 25,685 bp each), a large single-copy region (LSC, 86,525 bp), and a small single-copy region (SSC, 17,367 bp). It encodes 132 genes, including 8 rRNA genes, 36 tRNAs genes, and 88 protein-coding genes. The GC content of *O. cooperi* cp genome is 37.8%. Phylogenetic analysis indicates that *O. cooperi* is close to *O. fragrans* in Oleaceae.

*Osmanthus cooperi* Hemsley, which belongs to the family Oleaceae, is an evergreen shrub or small tree native to eastern China. Because of its scented and beautiful flowers, this species has been brought into cultivation as ornamental and several cultivars have been reported (Xiang and Liu 2008). However, currently there is no information available regarding its genetic background. Chloroplast genomes are widely used in phylogeny (Yan et al. 2018), species conservation (Li et al. 2019), and studies of genome evolution (Dong et al. 2018). Here, we report the complete chloroplast genome sequence of *O. cooperi* based on next-generation sequencing. The annotated cp genome has been deposited into GenBank with the accession number MK841488.

Total genomic DNA was isolated from fresh leaves of an individual of *O. cooperi* from Nanjing Forestry University campus in Nanjing, China. The voucher specimen was deposited at the herbarium of Nanjing Forestry University (NF, accession number NF201550), and used for the subsequent shotgun library construction and the whole-genome sequencing on the Illumina NextSeq 500 Sequencing System (Illumina, CA, USA). The Illumina sequencing was conducted by Nanjing Genepioneer Biotechnologies Inc., Nanjing, China. Raw reads were trimmed using CLC Genomics Workbench v9 and the resultant clean reads were employed to assemble the cp genome using the program NOVOPlasty (Dierckxsens et al. 2017) and then annotated by CpGAVAS (Liu et al. 2012).

The cp genome of *O. cooperi* was 155,262 bp, with a typical quadripartite structure of the large (LSC, 86,525 bp) and small (SSC, 17,367 bp) single-copy regions, separated by a pair of inverted repeat regions (IRs, 25,685 bp). A total of 132 genes are encoded, including 88 protein-coding genes (80 PCG species), 36 tRNAs gene (29 tRNA species), and 8 rRNA genes (4 rRNA species). Most of the genes occurred in a single copy; however, eight protein-coding genes (*ndhB*, *rpl2*, *rpl23*, *rps7*, *rps12*, *ycf1*, *ycf2*, *ycf15*), seven tRNA genes (*trnA-UGC*, *trnI-CAU*, *trnI-GAU*, *trnL-CAA*, *trnN-GUU*, *trnR-ACG*, *trnV-GAC*), and four rRNA genes (*4.5S*, *5S*, *16S*, *23S*) are totally duplicated. A total of nine protein-coding genes (*atpF*, *ndhA*, *ndhB*, *rpl2*, *rpl16*, *rpoC1*, *rps12*, *rps16*, *petD*) contained one intron while the other two genes (*clpP*, *ycf3*) had two introns each. The overall GC content of *O. cooperi* genome is 37.8%, the same as some reported chloroplast genomes from the family Oleaceae (He et al. 2017; Duan et al. 2019), and the corresponding values in LSC, SSC, and IR regions are 35.8%, 32.0%, and 43.2%, respectively.

A maximum likelihood (ML) phylogenetic tree was reconstructed using the concatenated coding sequences of chloroplast PCGs for a panel of 30 species with the web server RAxML-HPC2 on TG ver. 7. 2. 8 on the Cipres web server (Miller et al. 2010). *Osmanthus cooperi* was clustered with other species in Oleaceae with 100% bootstrap values (Figure 1), and it was close to *O. fragrans*, the type species of *Osmanthus*. This study will provide a useful resource for studying the genetic diversity of *O. cooperi*, the phylogenetic relationships of the Oleaceae family, and conservation of this valuable species.

**Figure 1.**
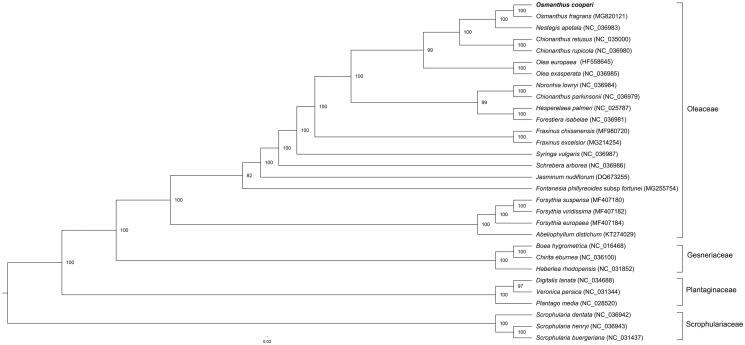
Maximum likelihood tree showing the relationship among *Osmanthus cooperi* and representative species within the order Lamiales. Shown next to the nodes are bootstrap support values based on 1000 replicates.

## References

[CIT0001] Dierckxsens N, Mardulyn P, Smits G 2017 NOVOPlasty: de novo assembly of organelle genomes from whole genome data. Nucleic Acids Res. 45:e18. 28204566 10.1093/nar/gkw955 PMC5389512

[CIT0002] Dong WL, Wang RN, Zhang NY, Fan WB, Fang MF, Li ZH 2018 Molecular evolution of chloroplast genomes of Orchid species: insights into phylogenetic relationship and adaptive evolution. IJMS. 19:716. 10.3390/ijms19030716 PMC5877577 29498674

[CIT0003] Duan YF, Li YF, Zhang C, Wang XR, Li MZ 2019 The complete chloroplast genome of sweet olive (*Osmanthus fragrans* Lour.). Mitochondrial DNA Part B. 4:1063–1064.

[CIT0004] He YX, Liu LX, Shuhan Yang SH, Meifang Dong MF, Yuan WJ, Shang FD 2017 Characterization of the complete chloroplast genome of Chinese fringetree (*Chionanthus retusus*). Conservation Genet Resour. 9:431–434.

[CIT0005] Li YF, Sylvester SP, Li M, Zhang C, Li X, Duan YF, Wang XR 2019 The complete plastid genome of *Magnolia zenii* and genetic comparison to Magnoliaceae species. Molecules. 24:261. 10.3390/molecules24020261 PMC6359370 30641990

[CIT0006] Liu C, Shi L, Zhu Y, Chen H, Zhang J, Lin X, Guan X 2012 CpGAVAS, an integrated web server for the annotation, visualization, analysis, and GenBank submission of completely sequenced chloroplast genome sequences. BMC Genomics. 13:715. 23256920 10.1186/1471-2164-13-715 PMC3543216

[CIT0007] Miller MA, Pfeiffer W, Schwartz T 2010 Creating the CIPRES Science Gateway for inference of large phylogenetic trees. Proceedings of the Gateway Computing Environments Workshop (GCE); Nov 14, New Orleans, LA.

[CIT0008] Xiang QB, Liu YL 2008 An illustrated monograph of the sweet Osmanthus cultivars in China. Hangzhou: Zhejiang Science and Technology Press.

[CIT0009] Yan MH, Fritsch PW, Moore MJ, Feng T, Meng AP, Yang J, Deng T, Zhao CX, Yao XH, Sun H, Wang HC 2018 Plastid phylogenomics resolves infrafamilial relationships of the Styracaceae and sheds light on the backbone relationships of the Ericales. Mol Phylogenet Evol. 121:198–211. 29360618 10.1016/j.ympev.2018.01.004

